# Differential glucocorticoid metabolism in patients with persistent versus resolving inflammatory arthritis

**DOI:** 10.1186/s13075-015-0633-2

**Published:** 2015-05-14

**Authors:** Dominika E Nanus, Andrew D Filer, Lorraine Yeo, Dagmar Scheel-Toellner, Rowan Hardy, Gareth G Lavery, Paul M Stewart, Christopher D Buckley, Jeremy W Tomlinson, Mark S Cooper, Karim Raza

**Affiliations:** Rheumatology Research Group, University of Birmingham, Edgbaston, Birmingham B15 2TT UK; Centre for Endocrinology, Diabetes and Metabolism, University of Birmingham, Edgbaston, Birmingham B15 2TT UK; Rheumatology, University Hospitals Birmingham NHS Foundation Trust, Edgbaston, Birmingham B15 2TH UK; Faculty of Medicine and Health, University of Leeds, Worsley Building, Leeds, LS2 9JT UK; Rheumatology, Sandwell and West Birmingham Hospitals NHS Trust, Dudley Road, Birmingham, B18 7QH UK; Oxford Centre for Diabetes, Endocrinology and Metabolism, University of Oxford, Churchill Hospital, Old Road, Headington, Oxford, OX3 7LE UK; ANZAC Research Institute, Concord Repatriation General Hospital, University of Sydney, Hospital Road, Sydney, NSW 2139 Australia

## Abstract

**Introduction:**

Impairment in the ability of the inflamed synovium to generate cortisol has been proposed to be a factor in the persistence and severity of inflammatory arthritis. In the inflamed synovium, cortisol is generated from cortisone by the 11β-hydroxysteroid dehydrogenase type 1 (11β-HSD1) enzyme. The objective of this study was to determine the role of endogenous glucocorticoid metabolism in the development of persistent inflammatory arthritis.

**Methods:**

Urine samples were collected from patients with early arthritis (symptoms ≤12 weeks duration) whose final diagnostic outcomes were established after clinical follow-up and from patients with established rheumatoid arthritis (RA). All patients were free of disease-modifying anti-rheumatic drugs at the time of sample collection. Systemic measures of glucocorticoid metabolism were assessed in the urine samples by gas chromatography/mass spectrometry. Clinical data including CRP and ESR were also collected at baseline.

**Results:**

Systemic measures of 11β-HSD1 activity were significantly higher in patients with early arthritis whose disease went on to persist, and also in the subgroup of patients with persistent disease who developed RA, when compared with patients whose synovitis resolved over time. We observed a significant positive correlation between systemic 11β-HSD1 activity and ESR/CRP in patients with established RA but not in any of the early arthritis patients group.

**Conclusions:**

The present study demonstrates that patients with a new onset of synovitis whose disease subsequently resolved had significantly lower levels of systemic 11β-HSD1 activity when compared with patients whose synovitis developed into RA or other forms of persistent arthritis. Low absolute levels of 11β-HSD1 activity do not therefore appear to be a major contributor to the development of RA and it is possible that a high total body 11β-HSD1 activity during early arthritis may reduce the probability of disease resolution.

**Electronic supplementary material:**

The online version of this article (doi:10.1186/s13075-015-0633-2) contains supplementary material, which is available to authorized users.

## Introduction

Glucocorticoids (GCs) regulate the body’s response to stress and inflammation, glucose metabolism and many other physiological processes. Secretion of endogenous GCs from the adrenal cortex is controlled by the hypothalamic-pituitary-adrenal (HPA) axis. In contrast to exogenous GCs, which are widely prescribed for the treatment of chronic inflammatory conditions, the role of endogenous GCs in the development and resolution of inflammation is less well characterised. Inadequate endogenous GC synthesis during inflammation has been proposed as an aetiological factor in the development of rheumatoid arthritis (RA). The link between endogenous GCs and the development of RA was initially thought due to defects in the HPA axis [1]. This hypothesis has been supported by clinical trials in which early GC treatment has reduced the risk of patients with early inflammatory arthritis developing persistent disease [2]. It is now known that GC action at a tissue level can also be regulated by local metabolism of GCs [3]. It has been previously demonstrated that inflamed synovial tissue in patients with RA can interconvert inactive and active GCs through the expression of the 11β-hydroxysteroid dehydrogenase enzymes (11β-HSDs) [4,5]. The two primary isoenzymes found in humans are 11β-HSD1, a bidirectional enzyme that primarily converts hormonally inactive cortisone into hormonally active cortisol, and 11β-HSD2 that solely inactivates cortisol to cortisone [6].

The GC-activating capacity of synovial tissue explants (a reaction carried out by 11β-HSD1) has been shown to directly correlate with histological and clinical measures of inflammation in patients with established RA [4,5]. These patients also have increased systemic measures of 11β-HSD1 activity that are significantly decreased upon treatment with anti-tumour necrosis factor alpha (TNFα) therapy [7]. This suggests that the observed upregulation of 11β-HSD1 in established RA can, at least partially, be explained by inflammation.

Despite there being a clear link between disease activity in established RA and activation of glucocorticoids by 11β-HSD1 within synovial tissue, it has been proposed that there is a ‘relative’ deficiency in the amount of cortisol synthesised in this condition compared to patients with osteoarthritis [4,8]. This deficiency in cortisol production could predispose to the persistence of inflammation. These observations were, however, made in patients with long-standing RA and the relationship between 11β-HSD1 activity and inflammation in patients with early untreated inflammatory arthritis has not been explored.

Previous work thus raises the possibility that patients with early arthritis are predisposed to develop persistent arthritis if they have a deficiency in local 11β-HSD1-mediated GC production. In this study, we examined total body levels of 11β-HSD1 activity in a cohort of patients with early inflammatory arthritis and, in particular, examined the relationship between enzyme activity and the subsequent outcome of inflammatory arthritis.

## Methods

### Patients

Patients (all aged over 18) were part of the Birmingham Early Arthritis Cohort (BEACON) and were recruited from Sandwell and West Birmingham Hospitals NHS Trust and University Hospitals Birmingham NHS Foundation Trust.

All patients with early arthritis presented with clinically apparent synovial swelling and a symptom duration of 12 weeks or less at the time of recruitment. The final diagnostic outcome was determined after 18 months of clinical follow-up when patients were assigned to one of the following three outcome groups: (1) persistent inflammatory arthritis that did not fulfil the 1987 American College of Rheumatology (ACR) classification criteria for RA [9]; (2) persistent RA, according to the 1987 ACR classification criteria [9]; (3) resolving inflammatory arthritis. Patients were classified as having a resolving inflammatory arthritis if they had no clinically apparent joint swelling at final follow-up, were not receiving disease-modifying drugs or steroids and had not received such drugs in the previous three months.

In addition, patients who fulfilled the 1987 ACR classification criteria for RA and had a symptom duration of more than 12 weeks at initial assessment were recruited as patients with ‘established RA’.

No patients in the current study received either glucocorticoid or disease-modifying anti-rheumatic drug therapy prior to recruitment and the collection of baseline urine or blood samples.

Clinical data including tender and swollen joint counts were collected, C-reactive protein (CRP) and erythrocyte sedimentation rate (ESR) measured, and the 28-item disease activity score (DAS28) using ESR scores calculated at the first appointment. Urine samples obtained from a single void were collected in a standardised manner in the mid-morning from all patients with early arthritis and established RA at the first appointment, snap frozen, and stored at -80°C. The study was conducted in compliance with the Helsinki declaration and was approved by the South Birmingham Local Research Ethics Committee. All subjects gave written informed consent.

### Measurement of urinary corticosteroid metabolites

Urinary corticosteroid metabolite levels were measured by gas chromatography/mass spectrometry (GC/MS) using a 5970 mass spectrometer (Hewlett-Packard, Houston, TX, USA) and a previously described method [10]. Systemic (that is total body) measures of 11β-HSD1 activity were calculated as the urinary (tetrahydrocortisol + 5αtetrahydrocorticol)/tetrahydrocortisone ((THF + 5αTHF)/THE) ratio and the cortols/cortolones ratio. The urinary-free cortisol/cortisone ratio (UFF/UFE) was also measured, being a sensitive indicator of changes in renal 11β-HSD2 activity. As an additional control, the activity of another enzyme system that metabolises cortisol, 5α-reductase, was measured and was calculated as the urinary 5αTHF/THF ratio. To determine whether these steroid ratios in a mid-morning spot urine sample were representative of complete 24-hour urine samples, we collected mid-morning spot urine samples from six healthy volunteers and compared results obtained from these with results from total 24-hour urine collections obtained from the same individuals. We found that the (THF + 5αTHF)/THE) ratio measured in these spot urine samples were highly correlated (r = 0.9590, *P* = 0.0025) with those obtained on a 24-hour sample (Figure S1 in Additional file 1). This confirmed that the measure of 11β-HSD1 activity obtained via a mid-morning urine sample accurately reflected daily 11β-HSD1 activity. Therefore for practical purposes, we used mid-morning spot urine samples to measure indices of glucocorticoid metabolism in the present study.

### Statistics

All statistical tests were performed using GraphPad Prism5, (GraphPad Software, San Diego, CA, USA). Normality of distribution of urine corticosteroid metabolite ratios were examined by the Kolmogorov-Smirnov test. When differences between different outcome groups were compared, if the data were normally distributed the unpaired *t* test was used. For non-normally distributed variables the Mann-Whitney test was used. Given the exploratory nature of our analyses, the reported *P* values were not adjusted for multiple comparisons. A *P* value <0.05 was considered statistically significant. Correlation analysis was performed using the Spearman’s test.

## Results

### Systemic measures of GC metabolism in patients with early arthritis in relation to clinical outcome and in patients with established RA

In total, 75 patients were recruited to the study and had full clinical assessment and urinary corticosteroid analysis. Fifty-five patients had early arthritis (symptom duration <12 weeks at initial recruitment) and 20 had established RA (fulfilling 1987 ACR classification criteria and having a symptom duration of >12 weeks at initial recruitment). Of the 55 early arthritis patients 31 subsequently developed persistent inflammatory arthritis (referred to as ‘early persistent arthritis (all)’). Of these 31 patients, 18 fulfilled classification criteria for RA (referred to as ‘early persistent RA’). Twenty-four of the 55 patients with early arthritis had a resolving disease course (referred to as ‘early resolving arthritis’). Demographic and clinical details of participants are shown in Table 1.

**Table 1:** **Demographic and clinical variables for patient with early arthritis and established RA**

**Outcome group**	**Early resolving arthritis**	**Early persistent arthritis (All)**	**Early persistent RA**	**Established RA**	***P***
Number	24	31	18	20	na
Female	16 (67%)	21 (68%)	12 (67%)	15 (75%)	ns
n (%)					
Age, years; mean (SD)	51.8 (14.4)	56.9 (18.2)	60.72 (15.8)	58.2 (14.3)	ns
CRP, mg/l;	8 (0-14)	25 (10-44)	27 (10.75-68)	9 (0-44.75)	*P* = 0.0221 Early p arthritis (all) vs early resolving arthritis
Median (IQR)					*P* = 0.0184 Early p RA vs early resolving arthritis
ESR, mm/h;	20.5 (12-44.75)	26 (20-60.75)	24 (19.75-60.75)	25 (9.75-48.75)	ns
Median (IQR)					
DAS28, ESR;	3.7 (3.5-5.1)	5.3 (4.4-6.7)	6.1 (4.25-6.8)	5.3 (4.3-6.1)	*P* = 0.0013 Early p arthritis (all) vs early resolving arthritis
Median (IQR)					*P* = 0.0054 Early p RA vs early resolving arthritis
Disease duration, weeks;	6.5 (4-9.5)	8 (6-10)	8 (5.75-10)	42 (22.25-82.5)	*P* < 0.0001 Established RA vs early p RA
Median (IQR)					
Tender joint count 28;	2.5 (1-4.75)	7 (2-17)	8.5 (2.75-15.5)	8.5 (4.25-19.75)	*P* = 0.0072 Early p arthritis (all) vs early resolving arthritis
Median (IQR)					*P* = 0.0072 Early p RA vs early resolving arthritis
Swollen joint count 28;	1 (1-3.5)	4 (1-11)	6.5 (3-13)	7 (4-13.5)	*P* = 0.0034 Early p arthritis (all) vs early resolving arthritis
Median (IQR)					*P* = 0.0001 Early p RA vs early resolving arthritis
RF + ve	3 (12.5%)	10 (32%)	9 (50%)	8 (40%)	ns
n (%)					
Anti-CCP antibodies	0 (0%)	9 (29%)	8 (44%)	11 (55%)	*P* = 0.0004
n (%)					

Early arthritis patients were assigned to one of the following three outcome groups after clinical follow-up: resolving arthritis (column 2), persistent arthritis (column 3) and persistent RA (column 4; a subgroup of the persistent arthritis patients). In addition, data are shown for patients who presented with established RA (column 5). Data shown for normally distributed variables are mean (SD) values and for non-normally distributed variables are median (IQR) values. Comparisons between different outcome groups for normally distributed clinical characteristics were carried out using the unpaired *t* test and comparison between different outcome groups for non-normally distributed clinical characteristics were carried out using the Mann-Whitney test (early persistent arthritis (all) vs early resolving arthritis; early persistent RA vs early resolving arthritis; early persistent RA vs established RA). Comparisons between different outcome groups for gender and autoantibody status were carried out using the chi-square test. A *P* value <0.05 was considered statistically significant. RA, rheumatoid arthritis; na, non applicable; ns, non significant; SD, standard deviation; CRP, C-reactive protein; IQR, interquartile range; ESR, erythrocyte sedimentation rate; DAS28, ESR, 28-item disease activity score calculated using ESR; RF, rheumatoid factor; anti-CCP antibodies, anti-cyclic citrullinated peptide antibodies.

**Figure 1:**
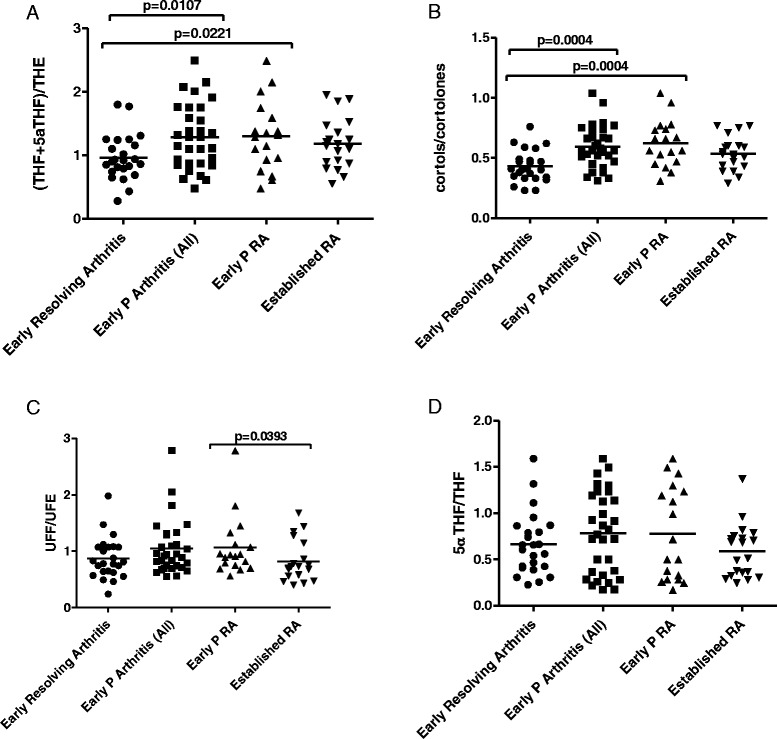
Differences in systemic measures of GC metabolism in patients with early arthritis and established RA. Systemic measures of 11β-HSD1 activity in patients with early arthritis of different outcomes and established RA: (THF + 5αTHF)/THE ratio **(A)** and cortols/cortolones ratio **(B)**. UFF/UFE ratio, an indicator of changes in renal 11β-HSD2 activity **(C)**. 5αTHF/THF, a systemic measure of 5α-reductase activity **(D)**. Values for (THF + 5αTHF)/THE ratio, cortols/cortolones ratio and 5αTHF/THF ratio were normally distributed whereas values for UFF/UFE ratio were not normally distributed. Individual values and means are represented on the graph. Comparisons between different outcome groups for normally distributed urine corticosteroid metabolite ratios ((THF + 5αTHF)/THE and cortols/cortolones) were carried out using the unpaired *t* test. Comparisons between different outcome groups for non-normally distributed urine corticosteroid metabolite ratios (UFF/UFE and 5αTHF/THF) were carried out using the Mann-Whitney test. A *P* value <0.05 was considered statistically significant. Patients with early resolving arthritis (circles), all patients with early persistent arthritis (squares), early persistent RA (upward triangles) and established RA (downward triangles). 11β-HSD, 11β-hydroxysteroid dehydrogenase type 1; GC, glucocorticoids; RA, rheumatoid arthritis; (THF + 5αTHF)/THE ratio, (tetrahydrocortisol + 5αtetrahydrocorticol)/tetrahydrocortisone ratio; UFF/UFE ratio, urinary-free cortisol/cortisone ratio.

Systemic measures of 11β-HSD1 activity were assessed and compared between the different outcomes in subjects with early arthritis and also between the patients with early persistent RA and patients with established RA. Patients with early persistent arthritis and the subgroup of patients with early persistent RA had a significantly higher mean level of the THF + 5αTHF/THE ratio when compared with patients in whom synovitis subsequently resolved (Figure 1A). 11β-HSD1 activity in patients with established RA was not significantly different to that in the early persistent RA group. Examination of cortols/cortolones ratios as an additional measure of 11β-HSD1 activity supported these findings, being significantly greater in the early persistent arthritis and early persistent RA groups compared to patients whose synovitis subsequently resolved (Figure 1B). In addition, there was no significant difference in this measure between early persistent RA patients and established RA patients. There was no significant difference in the UFF/UFE ratio (an indicator of renal 11β-HSD2 activity) between early resolving arthritis patients and patients who subsequently developed RA or other forms of persistent arthritis (Figure 1C). However, there was a significant difference between early persistent RA and established RA.

There was no significant difference between the outcome groups in 5αTHF/THF ratio, a measure of 5α-reductase activity (Figure 1D).

### Clinical measures of disease activity in patients with early arthritis and established RA

**Figure 2:**
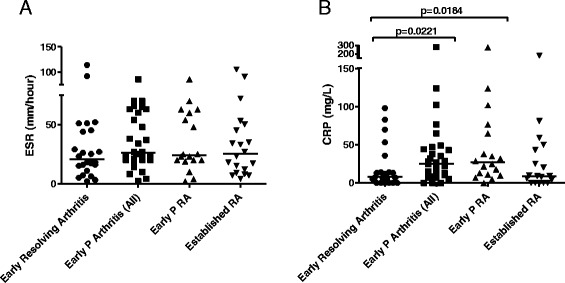
Clinical measures of disease activity in patients with early arthritis and established RA. ESR **(A)** and CRP **(B)** levels in patients with early resolving arthritis (circles), all patients with early persistent arthritis (squares), early persistent RA (upward triangles) and established RA (downward triangles). Individual values and medians are represented on the graph. Data for ESR and CRP were not normally distributed; the Mann-Whitney test was used for comparison between the outcome groups. A *P* value <0.05 was considered statistically significant. ESR, erythrocyte sedimentation rate (mm/hour); CRP, C-reactive protein (mg/L). RA, rheumatoid arthritis.

Inflammation is known to influence 11β-HSD1 activity *in vivo* [7]. To understand the possible influence of levels of inflammation on the observed differences in global 11β-HSD1 activity between different groups of early arthritis patients, we compared the levels of ESR and CRP in these groups (Figure 2A-B). Levels of ESR were not significantly different between different outcome groups (Figure 2A). However, the baseline level of CRP in patients with early synovitis that persisted was higher than that in patients whose synovitis resolved (Figure 2B).

### Relationship between inflammation and 11β-HSD1 activity in patients with early arthritis and established RA

**Figure 3:**
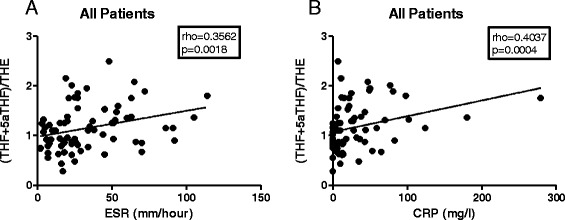
Correlation of ESR and CRP with global 11β-HSD1 activity in all patients analysed together. Correlation between (THF + 5αTHF)/THE ratio and ESR **(A)**, and CRP **(B)**. Correlation coefficient (rho) and the significance of the correlation (*P* value) were calculated using Spearman’s correlation. ESR, erythrocyte sedimentation rate (mm/hour); CRP, C-reactive protein (mg/L). 11β-HSD, 11β-hydroxysteroid dehydrogenase type 1; (THF + 5αTHF)/THE ratio, (tetrahydrocortisol + 5αtetrahydrocorticol)/tetrahydrocortisone ratio.

**Figure 4:**
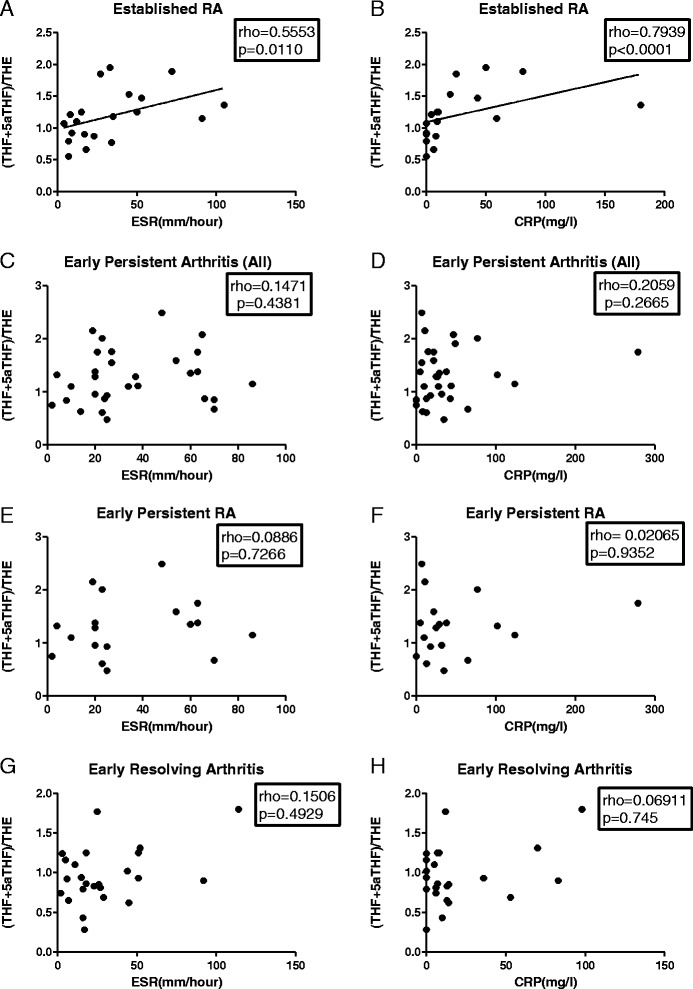
Correlation of ESR and CRP with global 11β-HSD1 activity in patients with early arthritis and established RA analysed individually. Correlation between (THF + 5αTHF)/THE ratios and: ESR in established RA patients **(A)**, CRP in established RA patients **(B)**, ESR in all patients with early persistent arthritis **(C)**, CRP in all patients with early persistent arthritis **(D)**, ESR in early persistent RA patients **(E)**, CRP in early persistent RA patients **(F)**, ESR in early resolving arthritis patients **(G)** and CRP in early resolving arthritis patients **(H)**. Correlation coefficient (rho) and the significance of the correlation (*P* value) were calculated using Spearman’s correlation. ESR, erythrocyte sedimentation rate (mm/hour); CRP, C-reactive protein (mg/L). 11β-HSD, 11β-hydroxysteroid dehydrogenase type 1; CRP, C-reactive protein (mg/L); RA, rheumatoid arthritis.

**Figure 5:**
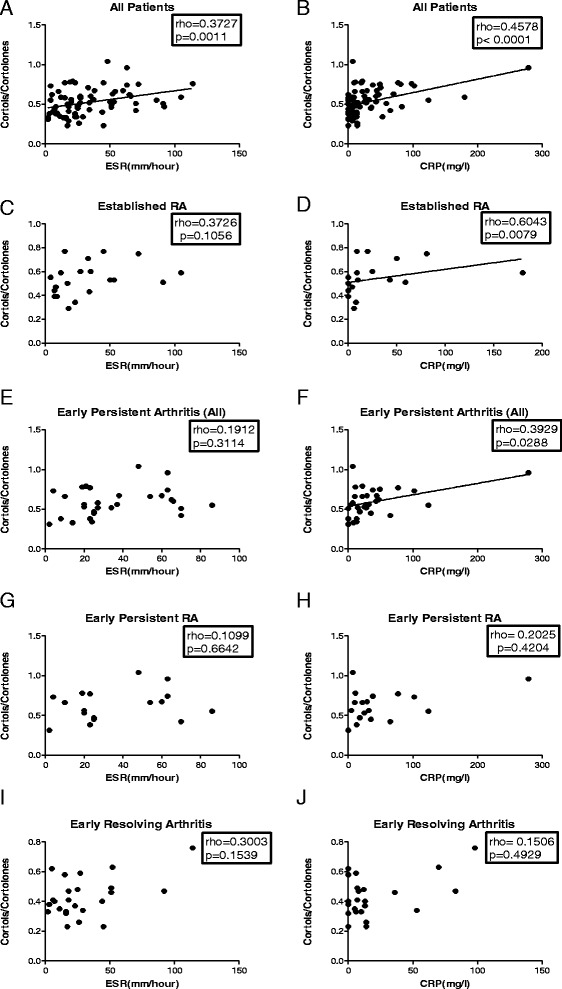
Correlation of ESR and CRP with global 11β-HSD1 activity in patients with early arthritis and established RA analysed individually. Correlation between cortols/cortolones ratio and: ESR in all recruited patients analysed together **(A)**, CRP in all recruited patients analysed together **(B)**, ESR in established RA patients **(C)**, CRP in established RA patients **(D)**, ESR in all patients with early persistent arthritis **(E)**, CRP in all patients with early persistent arthritis **(F)**, ESR in early persistent RA patients **(G)**, CRP in early persistent RA patients **(H)**, ESR in early resolving arthritis patients **(I)**, CRP in early resolving arthritis patients **(J)**. Correlation coefficient (rho) and the significance of the correlation (*P* value) were calculated using Spearman’s correlation. ESR, erythrocyte sedimentation rate (mm/hour); CRP, C-reactive protein (mg/L). 11β-HSD, 11β-hydroxysteroid dehydrogenase type 1; RA, rheumatoid arthritis.

We assessed the relationship between 11β-HSD1 activity and ESR/CRP in the different disease groups. The level of global 11β-HSD1 activity, measured as the (THF + 5αTHF)/ THE ratio, correlated significantly with ESR and CRP when all patients were analysed together (Figure 3A-B). Similarly, a significant positive correlation was observed in patients with established RA, where patients with the greatest increase in the ESR (Figure 4A) and in CRP (Figure 4B) had also greatest increase in the (THF + 5αTHF)/THE ratio. In contrast, there were no statistically significant correlations between ESR or CRP and systemic 11β-HSD1 activity in any of the early arthritis patients group (Figure 4C-H). Correlations between cortols/cortolones ratio and ESR/CRP in different outcome groups followed a pattern similar to the (THF + 5αTHF)/THE data and are presented in Figure 5.

## Discussion

This study shows that patients with a new onset of synovitis whose disease subsequently resolved had significantly lower levels of systemic 11β-HSD1 activity at presentation when compared with patients whose synovitis persisted. This observation appears contrary to that predicted on the basis of previous work examining 11β-HSD1 activity in patients with established rheumatic diseases. Specifically, these data do not support the hypothesis that the development of persistent arthritis is associated with a defect in local GC production via reduced 11β-HSD1 activity at the earliest stages of clinically apparent disease. By contrast, these data raise the possibility that a high total body 11β-HSD1 activity during early arthritis may reduce the probability of disease resolution.

Our previous work has demonstrated that both systemic measures of 11β-HSD1 activity and also local GC activation within the synovium of patients with established RA are related to the extent of inflammation as measured by the ESR [5]. In the current study, we did not observe a significant difference in the ESR levels measured between patients with early arthritis of different outcomes. However, we did observe a significant difference in the level of CRP. Patients with early persistent arthritis had significantly higher levels of CRP than patients with resolving synovitis, mirroring the increase observed in systemic 11β-HSD1 activity in the early persistent group. It is possible that the difference in global 11β-HSD1 activity observed between patients with early persistent arthritis and patients with early resolving arthritis may be explained, in part, by a difference in the degree of inflammation. Certainly, *in vitro* experiments on mesenchymal cell population demonstrate that 11β-HSD1 is positively regulated by pro-inflammatory cytokines that are elevated in RA [11]. To better understand this phenomenon *in vivo*, we have investigated the relationship between inflammation and global 11β-HSD1 activity. When all patients were analysed together, we found a positive correlation between measures of inflammation and the GCs activating capacity of the 11β-HSD1 system (as previously described). Statistically, this relationship was even stronger in the group of patients with established RA. In contrast, we were not able to detect a statistically significant relationship between these parameters at an early stage of arthritis. The pathological processes governing the earliest clinically apparent phases of RA are not fully understood. It has been suggested that mechanisms operating at the onset of RA are distinct from those observed at the later phase of disease [12]. It is possible that differences in synovial pathology in the early and established phases of RA lead to different relationships between synovial inflammation and global 11β-HSD1 activity in the early and established phases of RA.

Based on the current findings, it is possible that high local production of active GCs may not be necessary for the resolution phase of early arthritis, and in fact that high total body 11β-HSD1 activity observed during early arthritis might be mechanistically linked to persistence. Glucocorticoids are powerful immunomodulators [13]. High doses of exogenous GCs are well known for their anti-inflammatory and immunosuppressive properties. However, it has recently been proposed that, in certain situations, endogenous GCs have a pro-inflammatory rather than anti-inflammatory effect [14]. For example, corticosteroids produced during exposure to an acute restraint stress can potentiate the pro-inflammatory response of microglia to lipopolysaccharide (LPS), whilst in the chronic collagen-induced arthritis (CIA) mouse model, 11β-HSD1 inhibition (using a chemical inhibitor) significantly attenuates inflammation [15,16]. This offers an explanation as to why synovitis persists in patients with a higher level of 11β-HSD1 activity, where high concentrations of endogenous GCs instead of contributing to resolution might paradoxically be driving the persistence of RA by augmenting inflammation. However, these observations are in contrast to findings in the acute K/BxN inflammatory arthritis model where global transgenic knock-down of 11β-HSD1 exacerbated both the intensity and duration of acute inflammation [17]. 11β-HSD1 thus appears to deliver contrasting actions in the context of different inflammatory arthritis models, with different observation in acute resolving and chronic persistent models.

An alternative explanation for our data is that local 11β-HSD1 activity is important for disease resolution in patients with early arthritis but that the extent of the elevation observed in patients with persistent disease is inadequate given the level of inflammation observed in these individuals. To fully dissect the role of endogenous GC metabolism in driving persistence versus resolution in patients with early arthritis, a clinical study would be required using an 11β-HSD1 inhibitor in patients with a new onset of inflammatory joint disease. The contrasting evidence based on different murine models of arthritis suggests that endogenous GCs might play different roles during the onset and persistence phases of RA.

The observed high levels of global 11β-HSD1 activity in the group of patients that went on to develop persistent arthritis, including RA, might contribute to some of the early extra-articular features of RA including early osteoporosis [18], muscle loss [19], and cardiovascular changes [20]. It was recently shown in animal models that 11β-HSD1 inhibition can be beneficial in attenuating atherosclerosis [21]. Therefore, targeting the 11β-HSD1 system could impact some of the extra-articular as well as articular manifestations of RA.

We have previously reported that synovial tissue 11β-HSD1 activity increases in response to inflammation and this tissue is the most likely source of the additional 11β-HSD1 activity seen in patients with inflammatory arthritis. However, this is not necessarily the only tissue that could influence global measures of 11β-HSD1 in patients with early arthritis. Using the techniques employed here, it was impossible to determine the tissue that contributed most to the global measures of 11β-HSD1 activity used in this study. At a tissue level the amount of GC exposure will depend on both local production and the contribution from the circulation (which is regulated by the HPA axis). The measures of 11β-HSD1 described here were based on mid-morning urine samples. The benefit of these measures is that they depend on ratios of metabolites as opposed to absolute levels of metabolites, which are known to change during the day. To confirm the validity of our approach we showed that mid-morning ratios closely reflected the 24-hour picture. It should also be emphasised that these measures of glucocorticoid metabolism are independent of changes in the HPA axis. However, for a complete evaluation of total tissue GC exposure (including components from circulating GCs and from locally generated GCs) additional studies would require multiple sampling of serum levels in association with 24-hour measurements of total cortisol metabolite excretion. This would allow an analysis of the systemic GC response in addition to the local tissue response described here and could give insight into whether there was a relationship between the local tissue response and the systemic, circulatory, response in these conditions.

## Conclusions

This study has, for the first time, examined the levels of global 11β-HSD1 activity in patients with early arthritis that subsequently either persisted or resolved. We found that patients with resolving synovitis had significantly lower levels of global 11β-HSD1 activity when compared with patients whose arthritis developed into RA or other form of persistent arthritis. The extent to which this increased level of global 11β-HSD1 activity observed in patients with persistent arthritis contributes to and/or drives persistence remains to be clarified.

## Additional file

Additional file 1: Figure S1. Correlation of the (THF + 5αTHF)/THE ratio of urinary glucocorticoid metabolites measured in 24-hour urine collections and in mid-morning spot urine samples of the same healthy individuals (n = 6). Correlation coefficient (r) and the significance of the correlation (*P* value) were calculated using Pearson correlation. A *P* value <0.05 was considered statistically significant.

## Abbreviations

(THF + 5αTHF)/THE ratio: (tetrahydrocortisol + 5αtetrahydrocorticol)/tetrahydrocortisone ratio
11β-HSDs: 11β-hydroxysteroid dehydrogenase enzymes
BEACON: Birmingham Early Arthritis Cohort
CIA: collagen-induced arthritis
CRP: C-reactive protein
DAS28: 28-item disease activity score
ESR: erythrocyte sedimentation rate
GC/MS: gas chromatography/ mass spectrometry
GCs: glucocorticoids
HPA axis: hypothalamic-pituitary-adrenal axis
LPS: lipopolysaccharide
RA: rheumatoid arthritis
TNFα: tumour necrosis factor alpha
UFF/UFE ratio: urinary-free cortisol/cortisone ratio
