# Supplementary material for: Differential glucocorticoid metabolism in patients with persistent versus resolving inflammatory arthritis
Source: Arthritis Res Ther. 2015 May 14;17(1):121. doi: 10.1186/s13075-015-0633-2 (PMC4431033; doi:10.1186/s13075-015-0633-2)

**Additional Figure 1. Correlation of the (THF+5αTHF)/THE ratio of urinary glucocorticoid metabolites measured in 24 hour urine collections and in mid-morning spot urine samples of the same healthy individuals (n=6).** Correlation coefficient (r) and the significance of the correlation (p value) were calculated using Pearson correlation. A p value <0.05 was considered statistically significant.

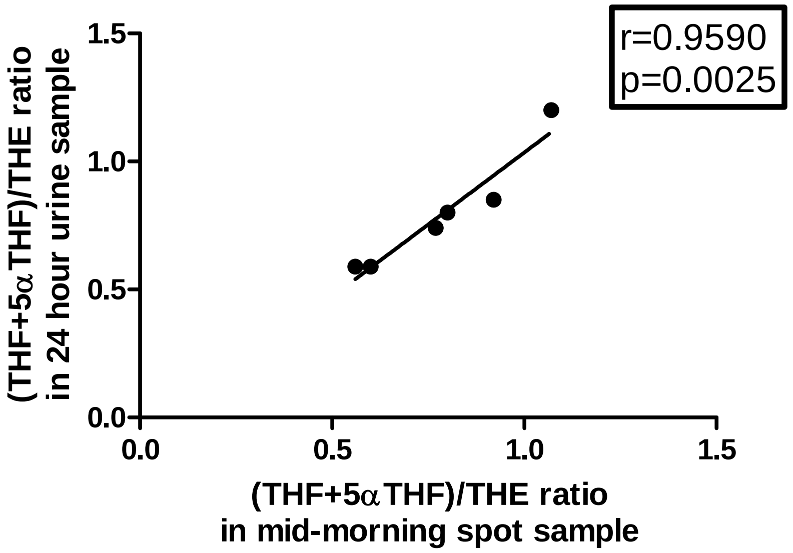

Supplement: Additional file 1: Figure S1. — Correlation of the (THF + 5αTHF)/THE ratio of urinary glucocorticoid metabolites measured in 24-hour urine collections and in mid-morning spot urine samples of the same healthy individuals (n = 6). Correlation coefficient (r) and the significance of the correlation (P value) were calculated using Pearson correlation. A P value <0.05 was considered statistically significant. [file 13075_2015_633_MOESM1_ESM.pdf]
